# Development and clinical application of a rapid IgM‐IgG combined antibody test for SARS‐CoV‐2 infection diagnosis

**DOI:** 10.1002/jmv.25727

**Published:** 2020-04-13

**Authors:** Zhengtu Li, Yongxiang Yi, Xiaomei Luo, Nian Xiong, Yang Liu, Shaoqiang Li, Ruilin Sun, Yanqun Wang, Bicheng Hu, Wei Chen, Yongchen Zhang, Jing Wang, Baofu Huang, Ye Lin, Jiasheng Yang, Wensheng Cai, Xuefeng Wang, Jing Cheng, Zhiqiang Chen, Kangjun Sun, Weimin Pan, Zhifei Zhan, Liyan Chen, Feng Ye

**Affiliations:** ^1^ State Key Laboratory of Respiratory Disease, National Clinical Research Center for Respiratory Disease Guangzhou Institute of Respiratory Health, The First Affiliated Hospital of Guangzhou Medical University Guangzhou China; ^2^ The 2nd Hospital of Nanjing Nanjing University of Chinese Medicine Nanjing China; ^3^ Chongqing Public Health Medical Center Chongqing China; ^4^ Union Hospital, Tongji Medical College Huazhong University of Science and Technology Wuhan China; ^5^ The 1st Affiliated Hospital of Nanchang University Nanchang China; ^6^ Department of Pulmonary and Critical Care Medicine Guangdong Second Provincial General Hospital Guangzhou Guangdong China; ^7^ Wuhan No. 1 Hospital Wuhan China; ^8^ The 1st Affiliated Hospital of Xi'an Jiaotong University Xi'an China; ^9^ Jiangsu Medomics Medical Technology Co., Ltd Nanjing China; ^10^ Hunan Provincial Center for Disease Control and Prevention Changsha China

**Keywords:** COVID‐19, fingerstick blood, lateral flow immunoassay, point‐of‐care testing, rapid IgM‐IgG combined test, SARS‐CoV‐2 virus infection

## Abstract

The outbreak of the novel coronavirus disease (COVID‐19) quickly spread all over China and to more than 20 other countries. Although the virus (severe acute respiratory syndrome coronavirus [SARS‐Cov‐2]) nucleic acid real‐time polymerase chain reaction (PCR) test has become the standard method for diagnosis of SARS‐CoV‐2 infection, these real‐time PCR test kits have many limitations. In addition, high false‐negative rates were reported. There is an urgent need for an accurate and rapid test method to quickly identify a large number of infected patients and asymptomatic carriers to prevent virus transmission and assure timely treatment of patients. We have developed a rapid and simple point‐of‐care lateral flow immunoassay that can detect immunoglobulin M (IgM) and IgG antibodies simultaneously against SARS‐CoV‐2 virus in human blood within 15 minutes which can detect patients at different infection stages. With this test kit, we carried out clinical studies to validate its clinical efficacy uses. The clinical detection sensitivity and specificity of this test were measured using blood samples collected from 397 PCR confirmed COVID‐19 patients and 128 negative patients at eight different clinical sites. The overall testing sensitivity was 88.66% and specificity was 90.63%. In addition, we evaluated clinical diagnosis results obtained from different types of venous and fingerstick blood samples. The results indicated great detection consistency among samples from fingerstick blood, serum and plasma of venous blood. The IgM‐IgG combined assay has better utility and sensitivity compared with a single IgM or IgG test. It can be used for the rapid screening of SARS‐CoV‐2 carriers, symptomatic or asymptomatic, in hospitals, clinics, and test laboratories.

## 1 INTRODUCTION

Since December 2019, a series of pneumonia cases of unknown cause emerged in Wuhan, Hubei, China, with clinical presentations greatly resembling viral pneumonia.^1^ Subsequently, pathogenic gene sequencing confirmed that the infected pathogen was a novel coronavirus, named 2019 novel coronavirus (SARS‐CoV‐2).^2^ Similar to previous outbreaks of coronavirus infection in humans, 2003 SARS‐CoV^3^, ^4^ and 2012 MERS‐CoV,^5^ SARS‐CoV‐2 infection caused the novel coronavirus disease (COVID‐19), its outbreak developed into an epidemic that quickly spread all over China and to more than 20 other countries.^6^ It has been listed as a public health emergency of international concern.^7^ The outbreak of this disease has caused the Chinese government to take drastic measures to contain the outbreak, including the quarantine of millions of residents in Wuhan and other affected cities. Countrywide interventions include delaying the resumption of workplaces, and encouraging citizens to stay and work from home, and so on.

However, these efforts are limited by one hard problem: how to differentiate the COVID‐19 cases from the healthy. For confirmed COVID‐19 cases, reported common clinical symptoms include fever, cough, myalgia, or fatigue.^8^ Yet these symptoms are not unique features of COVID‐19 because these symptoms are similar to that of other virus‐infected diseases such as influenza.^9^ Currently, virus nucleic acid real‐time polymerase chain reaction (RT‐PCR), CT imaging, and some hematology parameters are the primary tools for clinical diagnosis of the infection.^10^ Many laboratory test kits have been developed and used in testing patient specimens for COVID‐19 by Chinese CDC, US CDC, and other private companies. The virus nucleic acid RT‐PCR test has become the current standard diagnostic method for the diagnosis of COVID‐19. Yet these RT‐PCR test kits suffer from many limitations: (1) These tests have long turnaround times and are complicated in operation; they generally take on average over 2 to 3 hours to generate results. (2) The PCR tests require certified laboratories, expensive equipment, and trained technicians to operate. (3) There are some numbers of false negatives for RT‐PCR of COVID‐19.^11^ These limitations make RT‐PCR unsuitable for use in the field for rapid and simple diagnosis and screening of patients. It limits the outbreak containment effort. Therefore, there is an urgent need for a rapid, simple to use, sensitive, and accurate test to quickly identify infected patients of SARS‐CoV‐2 to prevent virus transmission and to assure timely treatment of patients.

Testing of specific antibodies of SARS‐CoV‐2 in patient blood is a good choice for rapid, simple, highly sensitive diagnosis of COVID‐19. It is widely accepted that immunoglobulin M (IgM) provides the first line of defense during viral infections, Before the generation of adaptive, high‐affinity IgG responses that are important for long term immunity and immunological memory.^12^ It was reported that after SARS infection, IgM antibody could be detected in patient blood after 3 to 6 days and IgG could be detected after 8 days.^13^, ^14^ Since COVID‐19 belongs to the same large family of viruses as those that cause the MERS and SARS outbreak, we assume its antibody generation process is similar, and detection of the IgG and IgM antibody against SARS‐CoV‐2 will be an indication of infection. Furthermore, detection of IgM antibodies tends to indicate recent exposure to SARS‐CoV‐2, whereas the detection of COVID‐19 IgG antibodies indicates virus exposure some time ago. Thus, we believe that the detection of both IgM and IgG could provide information on the virus infection time course. The rapid detection of both IgM and IgG antibodies will add value to the diagnosis and treatment of COVID‐19 disease.

Based on these, we developed a point‐of‐care lateral flow immunoassay (LFIA) test product, which can detect IgM and IgG simultaneously in human blood within 15 minutes. We tested the product in eight hospitals and Chinese CDC agencies to validate its clinical efficacy. The results demonstrated this rapid antibody test has high sensitivity and specificity. It can be used in hospitals, clinics, and testing laboratories. The test can also be effectively deployed in businesses, schools, airports, seaports and train stations, etc., giving it the potential to become a compelling force in the fight against this global threat.

## 2 MATERIALS AND METHODS

### 2.1 The materials for the manufacture of IgG‐IgM combined antibody test of COVID‐19

Anti‐human IgG and IgM (LF201001, LF201002) were purchased from Nanjing Lefushidai Inc, COVID‐19 recombinant antigen (MK201027) was developed and purified at Medomics. The recombinant antigen (MK201027) is receptor binding domain of SARS‐CoV‐2 Spike Protein, which is transient transfected in cell culture and purified by protein A affinity chromatography and size‐exclusion chromatography. The design of the antigen was based on the published SARS‐CoV‐2 sequence. Several different designs of antigen were tested and optimized. Eventually, MK201027 was picked into the testing product. Bovine serum albumin (BSA), and goat anti‐human IgG and IgM antibodies, rabbit IgG, and goat anti‐rabbit IgG antibodies were obtained from Sigma‐Aldrich. Forty‐nanometer gold nanoparticle (AuNP) colloids, NC membrane, and plastic pad were obtained from Shanghai KinBio Inc, the glass fiber conjugate pad was obtained from Whatman. The phosphate‐buffered saline (PBS) was purchased from Sigma‐Aldrich. Inactivated COVID‐19‐positive and ‐negative serum samples of patients were supplied by Hunan CDC, China.

### 2.2 Preparation of AuNP conjugates

To prepare the AuNP conjugate, SARS‐CoV‐2 recombinant protein dissolved in PBS (1 mg/mL) was added to the mixture of 1 mL AuNP colloid (40 nm in diameter, OD = 1) and 0.1 mL of borate buffer (0.1 M, pH 8.5). After incubation for 30 minutes at room temperature, 0.1 mL of 10 mg/mL BSA in PBS was added to the solution to block the AuNP surface. After incubation for 15 minutes at room temperature, the mixture was centrifuged at 10 000 rpm and 4°C for 20 minutes. The supernatant was discarded, and 1 mL of 1 mg/mL BSA in PBS was added to the AuNP conjugate to be resuspended. The centrifugation and suspension processes were repeated twice, and the final suspension solution was PBS. The AuNP‐rabbit IgG conjugates were prepared and purified by the same procedure.

### 2.3 Preparation of COVID‐19 rapid test of IgG‐IgM

The main body of the test strip consists of five parts, including plastic backing, sample pad, conjugate pad, absorbent pad, and NC membrane. Every component of the strip should be given a pretreatment described as follows: the NC membrane was attached to a plastic backing layer for cutting and handling. The anti‐human‐IgM, anti‐human‐IgG and anti‐rabbit‐IgG were immobilized at test M, G, and control line (C line),respectively. Conjugate pad was sprayed with mixture of AuNP‐COVID‐19 recombinant antigen conjugate and AuNP‐rabbit‐IgG. Sample pad was pretreated with BSA (3%, w/v) and Tween‐20 (0.5%, w/v) before use.

### 2.4 Testing of COVID‐19 samples using the LFIA system

#### 2.4.1 Patient and sample collection

The patients were recruited who conform to the diagnostic criteria of a suspected case of COVID‐19 according to the guidelines of diagnosis and treatment of COVID‐19^15^ including typical epidemiological history and clinical characteristics. These samples were collected from various hospitals and CDC testing laboratories (total eight) at six different provinces of China. The tests were conducted at the sites by clinical staff who followed the test procedure described in the product inserts. The respiratory tract specimen, including pharyngeal swab and sputum, was used to confirm COVID‐19 cases, and the blood, including serum and plasma, was used to test the IgM and IgG antibody.

#### 2.4.2 Sample testing

Before testing, the pouched device was opened immediately before use. When refrigerated blood samples were used for the test, they were warmed to room temperature (15°C‐30°C). During testing, 20 μL whole blood sample (or 10 μL of serum/plasma samples) was pipetted into the sample port followed by adding two to three drops (70‐100 μL) of dilution buffer (10 mM PBS buffer) to drive capillary action along the strip. The entire test took about 15 minutes to finish.

#### 2.4.3 Display of results

A total of three detection lines are on the stip. The control (C) line appears when the sample has flowed through the cartridge. The presence of anti‐SARS‐CoV‐2 IgM and anti‐SARS‐CoV‐2 IgG will be indicated by a red/pink test line in the M and G region. If only the control line (C) showed red, the sample is negative. Either M or G line or both lines turning into red indicates the presence of anti‐SARS‐CoV‐2‐IgM or anti‐SARS‐CoV‐2‐IgG or both antibodies in the specimen. If the control line does not appear red, the test is invalid, and the test should be repeated with another cartridge.

### 2.5 Data analysis

The rapid SARS‐CoV‐2 IgG‐IgM combined antibody test kits were tested at eight hospitals and Chinese CDC laboratories in different provinces, with a total of 397 clinical positive and 128 clinical negative patient blood samples. The test data was collected and analyzed. The specificity and sensitivity of the rapid test kits were calculated according to the following formulas:

Specificity(%)=100×[truenegative/(truenegative+falsepositive)],

Sensitivity(%)=100×[truepositive/(truepositive+falsenegative)].

## 3 RESULTS

### 3.1 Design and the finished product of SARS‐CoV‐2 rapid test of IgG‐IgM combined antibody kit

**Figure 1:**
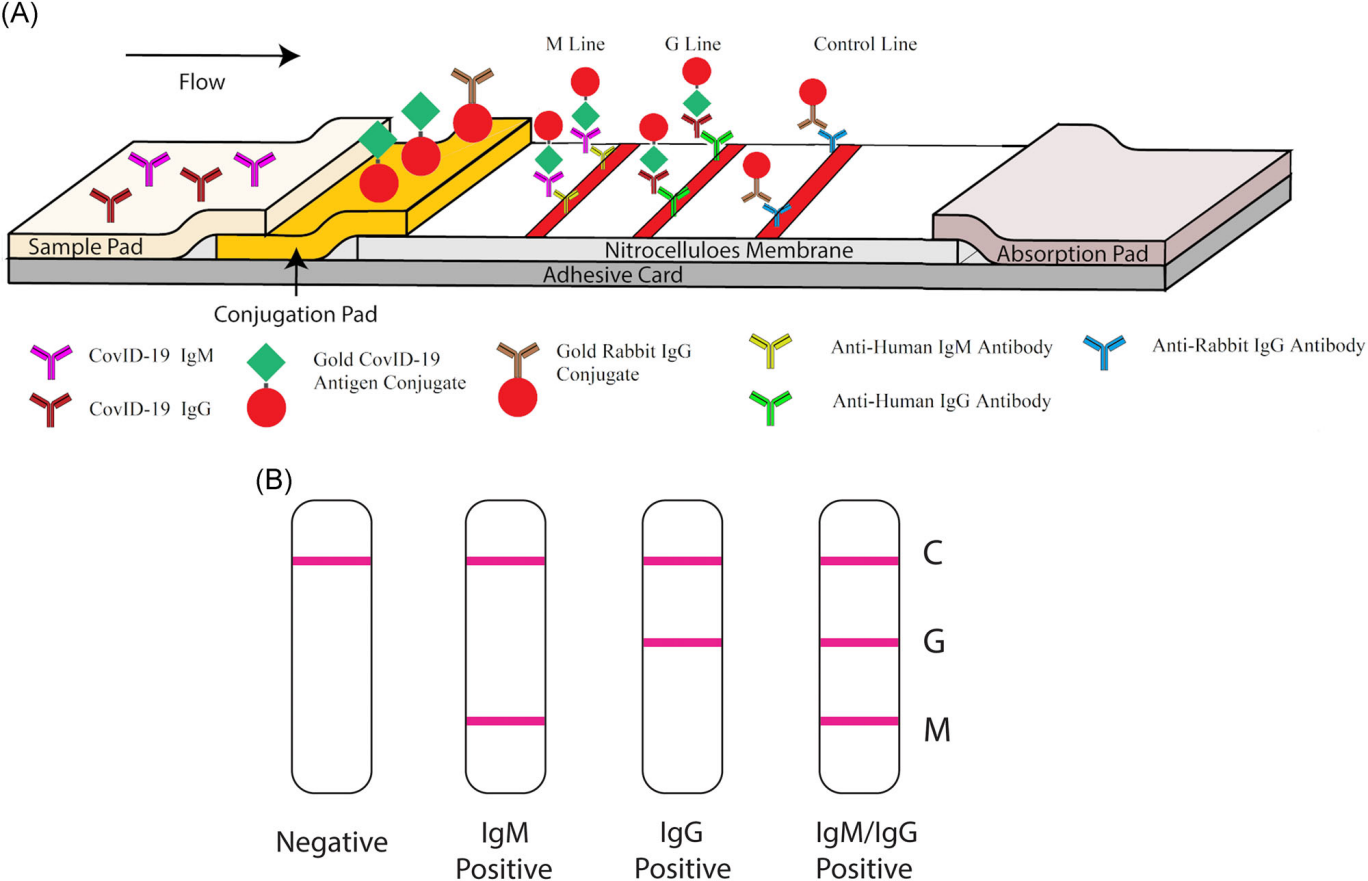
Schematic illustration of rapid SARS‐CoV‐2 IgM‐IgG combined antibody test. A, Schematic diagram of the detection device; B, an illustration of different testing results; C, means control line; G, means IgG line; M, means IgM line. IgG, immunoglobulin G; IgM, immunoglobulin M; SARS‐CoV‐2, severe acute respiratory syndrome coronavirus 2

The SARS‐CoV‐2 rapid IgG‐IgM combined antibody test kit is designed and manufactured by Jiangsu Medomics Medical Technologies, located in Nanjing, China. It is a lateral flow qualitative immunoassay for the rapid determination of the presence or absence of both anti‐SARS‐CoV‐2‐IgM and anti‐SARS‐CoV‐2‐IgG in human specimens (whole blood, serum, and plasma). The test kit comes with a test cartridge, sample dilution buffer, and a package insert. The testing cartridge has three detection bands, including a distal control band that appears when the sample has flowed to the end of the testing strip. The presence of SARS‐CoV‐2 IgG and IgM antibodies are indicated by a red/purple line in the specific region indicated on the device. The SARS‐CoV‐2 rapid IgG‐IgM combined antibody test strip, as shown in Figure 1, has two mouse anti‐human monoclonal antibodies (anti‐IgG and anti‐IgM) stripped on two separated test lines. A surface antigen from SARS‐CoV‐2 which can specifically bind to SARS‐CoV‐2 antibodies (including both IgM and IgG) is conjugated to colloidal gold nanoparticles and sprayed on conjugation pads. The AuNP‐rabbit IgG conjugates were also sprayed on conjugation pads for binding to anti‐rabbit IgG antibody which is immobilized on the control line (Figure 1A).

When testing, 10 to 15 μL specimen is added into the sample port followed by the addition of sample dilution buffer. The mechanism of the assay is based on the hydration and transport of reagents as they interact with the specimen across the strip via chromatographic lateral flow (Figure 1A). As the specimen flows through the device, anti‐SARS‐CoV‐2 IgG and IgM antibodies, if present in the specimen, are bound by the SARS‐CoV‐2 antigen labeled gold colorimetric reagent fixed on the conjugate pad. As the conjugated sample continues to travel up the strip, the anti‐ SARS‐CoV‐2 IgM antibodies are bound on the M(IgM) line, and the anti‐COVID‐19 IgG antibodies are bound to the G (IgG) line. If the specimen does not contain SARS‐CoV‐2 antibodies, no labeled complexes bind at the test zone and no lines could be observed. The remaining colloidal gold travels up the nitrocellulose to the control line zone, which captures the excess conjugate demonstrating that the fluid has migrated adequately through the device. A reddish‐purple line will appear at the control line zone during the performance of all valid tests whether the sample is positive or negative for SARS‐CoV‐2 infection. During the test, excess reagent including AuNP‐rabbit IgG conjugates migrate passes the control line zone, where the AnNP‐rabbit IgG conjugates bind to anti‐rabbit IgG to form a red line on the control line. Figure 1B is the illustration of different testing results reading for negative, IgM positive, IgG positive and IgM/IgG both positive situations.

**Figure 2:**
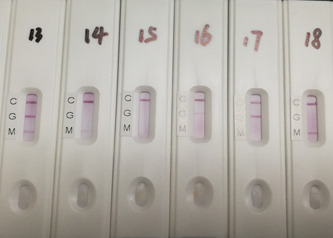
Representative photo for different patient blood testing results. (#13) Both IgM and IgG positive, (#14) IgM weak positive, (#15) Both IgM and IgG negative, (#16) IgG weak positive, (#17) IgG positive, and (#18) IgM positive. IgG, immunoglobulin G; IgM, immunoglobulin M

As one example to show real testing results, Figure 2 shows a testing result photo for six different test cartridges from six patients which represent several different types of results. In cartridge #13, the photo reading represents detection of both IgM and IgG; in cartridge #14 IgM only in low concentration; in cartridge #15, no IgM and IgG; in cartridge #16 IgG only in low concentration; in cartridge #17 IgG only in high concentration and in cartridge #18, IgM only in high concentration in patient bloods, respectively.

### 3.2 The detection sensitivity and specificity of SARS‐CoV‐2 IgG‐IgM combined antibody kit

To test the detection sensitivity and specificity of SARS‐CoV‐2 IgG‐IgM combined antibody test, blood samples were collected from COVID‐19 patients from multiple hospitals and Chinese CDC laboratories. The tests were done separately at each site. A total of 525 cases were tested: 397 (positive) clinically confirmed (including PCR test) SARS‐CoV‐2‐infected patients and 128 non‐SARS‐CoV‐2‐infected patients (128 negative). The testing results of vein blood without viral inactivation are summarized in Table 1. Of the 397 blood samples from SARS‐CoV‐2‐infected patients, 352 tested positive, resulting in a sensitivity of 88.66%. A total of 12 blood samples from the 128 non‐SARS‐CoV‐2 infection patients tested positive, generating a specificity of 90.63%. It was also found that 64.48% (256 of 397) of positive patients had both IgM and IgG antibodies (Table 1).

**Table 1:** The detection sensitivity and specificity of SARS‐CoV‐2 IgG‐IgM combined antibody reagent

	Clinical positive samples	Clinical negative samples
Sample quantity	397	128
IgG&IgM positive	256	1
IgG positive	24	1
IgM positive	72	10
Sensitivity	88.66%	
Specificity		90.63%

Abbreviations: IgG, immunoglobulin G; IgM, immunoglobulin M; SARS‐CoV‐2, severe acute respiratory syndrome coronavirus 2.

For the test, it is very important to know the data of infection time point from clinical samples which will be helpful to compare the data of single‐ or double‐positive in Table 1. Due to limited time, we do not have complete detailed information for how long each patient was infected or for how long each patient had symptoms when the blood sample was collected at all the clinical sites. We only had such data from one of the clinical site—Wuhan Red Cross Hospital. As a reference, by analyzing one subset data of 58 patients in Wuhan, it was found that 94.83% of the positive patients had both IgM‐ and IgG‐positive test lines, and 1.72%, 3.45% had only IgM or only IgG‐positive lines, respectively (Table 2). The test time was from day 8 to day 33 after infection symptoms appeared. Further studies and information collection are needed for this.

**Table 2:** IgM and IgG in positive patient blood samples from Wuhan Red Cross Hospital

	Number in positive samples	Percentage
IgM only	1	1.72
IgG only	2	3.45
Both IgM and IgG	55	94.83

Abbreviations: IgG, immunoglobulin G; IgM, immunoglobulin M.

### 3.3 SARS‐CoV‐2 IgG‐IgM combined antibody test in different types of blood samples

The above results have verified the sensitivity and specificity of kit detection in un‐inactivated vein blood. However, it is more convenient to collect fingerstick blood outside hospitals and clinics. To achieve a simpler operating process, we tested the performance of SARS‐CoV‐2 IgG‐IgM combined antibody kit with peripheral blood. Patient fingerstick blood and vein blood and plasma from the same patient were tested. As shown in Table 3, seven COVID‐19 patients and three healthy volunteers were recruited. We took the blood samples from fingerstick, serum of venous blood, and plasma of venous blood and tested them with the kits. Within the 7 patients, 3 patients have IgM only positive and 4 patients have both IgM and IgG positive. All healthy volunteers tested negative. The results showed that all of the positive and negative test results matched with 100% consistency among the corresponding blood samples. This result demonstrates that the SARS‐CoV‐2 IgG‐IgM combined antibody test kit can be used as a point‐of‐care test (POCT). It can be performed near the bedside with fingerstick blood.

**Table 3:** The evaluation of detection consistency in different types of blood samples

	COVID‐19 patients			Healthy person		
	Total samples	IgM Positive	IgG&IgM Positive	Total samples	IgM positive	IgG&IgM positive
Fingerstick blood	7	3	4	3	0	0
Serum of venous blood	7	3	4	3	0	0
Plasma of venous blood	7	3	4	3	0	0
Detection consistency	100%					

Abbreviations: IgG, immunoglobulin G; IgM, immunoglobulin M.

## 4 DISCUSSION

Here, we successfully developed a rapid IgG‐IgM combined antibody test kit for COVID‐19 diagnosis. The sensitivity and specificity of the kit were verified via the lab and clinical practice. This test kit provides a product to meet the urgent need for immunoassay tests in Chinese hospitals for the diagnosis of COVID‐19.

To make the kit suitable for different stages of the disease, we developed an IgG‐IgM combined antibody test for COVID‐19 infection (Figure 1). It was also been confirmed that the detection sensibility was higher in IgG‐IgM combined antibody test than in individual IgG or IgM antibody test (Table 1). Therefore, we more recommend the development of IgG‐IgM combined antibody test kits than the separate IgG or IgM antibody test kits, if there is a reliable technical system available. It is a better test for screening COVID‐19 patients.

This newly developed test kit, the IgG‐IgM combined antibody test kit, has a sensitivity of 88.66% and specificity of 90.63%. However, there were still false positive and false‐negative results (Table 1). The reasons for the false‐negative maybe, first, due to the low antibody concentrations. When IgM and IgG levels are below the detection limit (not determined yet) of this rapid test, the test results will be negative. Second, the difference in individual immune response antibody production could be one reason for the false‐negative results in COVID‐19 patients. The last but not least, IgM antibody will decrease and disappear after 2 weeks. In some cases, it is hard to know exactly when the patient was infected or how long the patient was infected. Thus, when the patient was tested, the IgM level might well be below its peak and not detectable by this test. Therefore, we encourage more research and development of the COVID‐19 IgG‐IgM combined antibody test kit to improve the diagnostic sensitivity and specificity for patients. Since COVID‐19 recombinant antigen was used in the test, our test is specific for COVID‐19 infection. The clinical testing data in this paper at different clinical sites from east, south, west, and middle of China confirmed the specificity of the test kit. However, because of the emergency of the outbreak of COVID‐19, we could not carry out normal research activities and perform enough tests to verify if there is interference from other IgM and IgG induced by different virus infections such as typical flu viruses. This study need to be done later.

This new rapid SARS‐CoV‐2 IgG‐IgM combined antibody test kit has several advantages. Compared to RT‐PCR, it saves time and it does not require equipment, it is simple to perform and only requires minimal training. It can be performed at the bedside, in any clinic or laboratory, at airports or at railway stations.^16^ It will be more convenient to use fingerstick blood or heel blood instead of vein blood for out‐of‐clinic screening. Our initial test results using fingertip blood were as good as that of vein blood (Table 3), which suggests that the SARS‐CoV‐2 IgG‐IgM combined antibody test kits can be developed as agents for rapid field detection. Another potential application of this test is screening asymptomatic SARS‐CoV‐2 carriers, it was reported that asymptomatic carriers could spread SARS‐CoV‐2 virus.^17^, ^18^ This finding made the current COVID‐19 outbreak control more difficult, because there is no method available to screen asymptomatic carriers. This rapid IgM‐IgG combined antibody test kit makes large‐scale screening of asymptomatic carriers possible. At least some if not all of the carriers are likely to have anti‐SARS‐CoV‐2 antibodies, as demonstrated by asymptomatic Zika virus carriers.^19^ Because this test can detect IgM and IgG simultaneously, it could be used for both early diagnosis (IgM) and for monitoring during treatment. SARS‐CoV‐2 infection starts at the lungs, not in the upper respiratory tract,^8^ therefore, sampling during the early infection stage using throat swab or sputum may not detect the virus. This is one possible explanation for high false negatives in the nucleic acid PCR test. However, this sampling effect should not have any effect on IgM and IgG detection with this rapid test.

Based on our knowledge and information, there are several other Chinese IVD companies developing or have developed similar products ranging from IgM only and IgM‐IgG combined tests. We do not know the details about their technical performance since there is no publication on them. We believe the good test products will be used in clinical sites and the information will emerge. We will carry out comparison studies later.

Certainly, this test cannot confirm virus presence, only provide evidence of recent infection, but it provides important immunological evidence for physicians to make the correct diagnosis along with other tests and to start treatment of patients. In addition, possible cross‐reactivity with other coronaviruses and flu viruses were not studied, and the change level of antibody was not compared in the different stages of SARS‐CoV‐2 infection. We believe the combination of nucleic acid RT‐PCR and the IgM‐IgG antibody test can provide more accurate SARS‐CoV‐2 infection diagnosis.

## 5 CONCLUSION

We developed a rapid SARS‐CoV‐2 IgG‐IgM combined antibody test using lateral flow immune assay techniques. It takes less than 15 minutes to generate results and determine whether there is a recent SARS‐CoV‐2 infection. It is easy to use, and no additional equipment is required. Results from this study demonstrated that this test is sensitive and specific. This rapid test has great potential benefits for the fast screening of SARS‐CoV‐2 infections, and it has already generated tremendous interest and increased clinical uses after a short time testing in Chinese hospitals.

## CONFLICT OF INTERESTS

The authors declare that there are no conflict of interests.

## References

[1] WHO . Novel coronavirus—China. http://wwwwhoint/csr/don/12‐january‐2020‐novel‐coronavirus‐china/en/. Accessed January 12, 2020.

[2] Zhu N , Zhang D , Wang W , et al. A novel coronavirus from patients with pneumonia in China, 2019. N Engl J Med. 2020;382:727‐733. 31978945 10.1056/NEJMoa2001017 PMC7092803

[3] Ksiazek TG , Erdman D , Goldsmith CS , et al. A novel coronavirus associated with severe acute respiratory syndrome. N Engl J Med. 2003;348(20):1953‐1966. 12690092 10.1056/NEJMoa030781

[4] Kuiken T , Fouchier RA , Schutten M , et al. Newly discovered coronavirus as the primary cause of severe acute respiratory syndrome. Lancet. 2003;362(9380):263‐270. 12892955 10.1016/S0140-6736(03)13967-0 PMC7112434

[5] Zaki AM , van Boheemen S , Bestebroer TM , Osterhaus AD , Fouchier RA . Isolation of a novel coronavirus from a man with pneumonia in Saudi Arabia. N Engl J Med. 2012;367(19):1814‐1820. 23075143 10.1056/NEJMoa1211721

[6] Centers for Disease Control and Prevention CfDCaP . Confirmed COVID‐19 Cases Globally. wwwcdcgov/coronavirus/COVID‐19/locations‐confirmed‐caseshtml#map. Accessed February 15, 2020.

[7] Prevention CCfDCa . Distribution of pneumonic outbreaks of COVID‐19 infection. http://2019ncovchinacdccn/COVID‐19/. Accessed February 19, 2020.

[8] Huang C , Wang Y , Li X , et al. Clinical features of patients infected with 2019 novel coronavirus in Wuhan, China. Lancet. 2020;395(10223):497‐506. 31986264 10.1016/S0140-6736(20)30183-5 PMC7159299

[9] Wang C , Yu H , Horby PW , et al. Comparison of patients hospitalized with influenza A subtypes H7N9, H5N1, and 2009 pandemic H1N1. Clin Infect Dis. 2014;58(8):1095‐1103. 24488975 10.1093/cid/ciu053 PMC3967826

[10] Jin YH , Cai L , Cheng ZS , et al. A rapid advice guideline for the diagnosis and treatment of 2019 novel coronavirus (COVID‐19) infected pneumonia (standard version). Mil Med Res. 2020;7(1):4. 32029004 10.1186/s40779-020-0233-6 PMC7003341

[11] Gallagher J . Are coronavirus tests flawed? BBC News . wwwbbccom/news/health‐51491763. Accessed 13 February, 2020.

[12] Racine R , Winslow GM . IgM in microbial infections: taken for granted? Immunol Lett. 2009;125(2):79‐85. 19539648 10.1016/j.imlet.2009.06.003 PMC2747358

[13] Lee HK , Lee BH , Seok SH , et al. Production of specific antibodies against SARS‐coronavirus nucleocapsid protein without cross reactivity with human coronaviruses 229E and OC43. J Vet Sci. 2010;11(2):165‐167. 20458159 10.4142/jvs.2010.11.2.165 PMC2873818

[14] Wan ZY , Zhang X , Yan XG . IFA in testing specific antibody of SARS coronavirus. South China J Prev Med. 2003;29(3):36‐37.

[15] Prevention CCfDCa . The guideline of diagnosis and treatment of COVID‐19. http://wwwchinacdccn/jkzt/crb/xcrxjb/202002/t20200209_212396html. Accessed February 9, 2020.

[16] Singer AJ , Williams J , Taylor M , Le Blanc D , Thode HC Jr. Comprehensive bedside point of care testing in critical ED patients: a before and after study. Am J Emerg Med. 2015;33(6):776‐780. 25836947 10.1016/j.ajem.2015.03.034

[17] Rothe C , Schunk M , Sothmann P , et al. Transmission of COVID‐19 Infection from an asymptomatic contact in Germany. N Engl J Med. 2020;382(10):970‐971. 32003551 10.1056/NEJMc2001468 PMC7120970

[18] Zou L , Ruan F , Huang M , et al. SARS‐CoV‐2 viral load in upper respiratory specimens of infected patients. N Engl J Med. 2020:NEJMc2001737. 10.1056/NEJMc2001737 PMC7121626 32074444

[19] Turner LH , Kinder JM , Wilburn A , et al. Preconceptual Zika virus asymptomatic infection protects against secondary prenatal infection. PLOS Pathog. 2017;13(11):e1006684. 29145516 10.1371/journal.ppat.1006684 PMC5689831

